# Dynamic spatio-temporal pruning for efficient spiking neural networks

**DOI:** 10.3389/fnins.2025.1545583

**Published:** 2025-03-25

**Authors:** Shuiping Gou, Jiahui Fu, Yu Sha, Zhen Cao, Zhang Guo, Jason K. Eshraghian, Ruimin Li, Licheng Jiao

**Affiliations:** ^1^Key Laboratory of Intelligent Perception and Image Understanding of Ministry of Education, School of Artificial Intelligence, Xidian University, Xi'an, China; ^2^Department of Electrical and Computer Engineering, University of California, Santa Cruz, Santa Cruz, CA, United States

**Keywords:** spiking neural networks, spatio-temporal pruning, dynamic vision sensor, sparse connectivity, adaptive temporal dynamics

## Abstract

Spiking neural networks (SNNs), which draw from biological neuron models, have the potential to improve the computational efficiency of artificial neural networks (ANNs) due to their event-driven nature and sparse data flow. SNNs rely on dynamical sparsity, in that neurons are trained to activate sparsely to minimize data communication. This is critical when accounting for hardware given the bandwidth limitations between memory and processor. Given that neurons are sparsely activated, weights are less frequently accessed, and potentially can be pruned to less performance degradation in a SNN compared to an equivalent ANN counterpart. Reducing the number of synaptic connections between neurons also relaxes memory demands for neuromorphic processors. In this paper, we propose a spatio-temporal pruning algorithm that dynamically adapts to reduce the temporal redundancy that often exists in SNNs when processing Dynamic Vision Sensor (DVS) datasets. Spatial pruning is executed based on both global parameter statistics and inter-layer parameter count and is shown to reduce model degradation under extreme sparsity. We provide an ablation study that isolates the various components of spatio-temporal pruning, and find that our approach achieves excellent performance across all datasets, with especially high performance on datasets with time-varying features. We achieved a 0.69% improvement on the DVS128 Gesture dataset, despite the common expectation that pruning typically degrades performance. Notably, this enhancement comes with an impressive 98.18% reduction in parameter space and a 50% reduction in time redundancy.

## 1 Introduction

Spiking Neural Networks (SNNs) are considered as the third generation of neural network models, and many recent studies aim to integrate spike-based processing from biological neurons with deep learning models with the aim of improving energy efficiency (Hassibi and Stork, 1992; Bohte et al., 2002; Roy et al., 2019). The human visual and auditory scenes can transduced into spike signals, a process that SNNs can better simulate in terms of reception and processing of this information (Eshraghian et al., 2021; Guo et al., 2023). When processed on neuromorphic hardware, neurons can be suppressed to prevent adding to data communication and computation demands, and SNNs are thus considered “event-driven” (Modaresi et al., 2023; Merolla et al., 2014; Davies et al., 2018). When compared to Artificial Neural Networks (ANNs), SNNs can leverage asynchronous, event-driven hardware, and with the right workloads, can drastically reduce the energy cost while retaining the ability to learn from temporal features (Xing et al., 2024). This advantage is currently widely applied in the research of neuromorphic computing algorithms and event data processing (Liu et al., 2024; Zhou and Zhang, 2021; Perez-Peña et al., 2013). However, SNNs most commonly target lightweight, edge-based applications while the demands of neural networks far exceed what many neuromorphic accelerators are capable of processing. For example, Tianjic can process models with approximately 40,000 neurons and 10 million synapses (Pei et al., 2019; Deng et al., 2020), whereas a VGG-16 architecture requires 14 million synapses and 280,000 neurons to run the CIFAR-10 dataset. While there is ongoing research to build scalable, server-based neuromorphic systems (Gonzalez et al., 2024; Vogginger et al., 2024; Orchard et al., 2021), developing compression methods is crucial to reduce the burden of inter-chip data communication: one of the challenges SNNs are aiming to solve.

Pruning is a fundamental method for model compression (Janowsky, 1989; Chauvin, 1988). In neuromorphic computing, pruning is particularly beneficial as it reduces computational load and memory access, addressing hardware constraints such as bandwidth and energy efficiency. Previous pruning practices in ANNs can be categorized based on the scale of pruning, which includes Filter-level, Group-level, and Connection-level pruning (Gao et al., 2021). Pruning at the filter level is structured but tends to have a considerable impact on model performance. For example, the scaling factors of Batch Normalization layers have been linked with synaptic connection strengths, enabling the quantification and subsequent pruning of filters (Lin et al., 2018; Meng et al., 2023; Li et al., 2024). Connection-level pruning often results in less accuracy degradation and high sparsity, however, the sparsity is unstructured. For instance, pruning connections based on the magnitude of their L1-norm weights, where connections with magnitudes below a certain threshold are pruned (Chauvin, 1988), has been effectively utilized in later works (Gale et al., 2019; Han et al., 2015; Renda et al., 2020), including implementations in the SNN domain (Kim et al., 2022; Shi et al., 2019). However, unstructured sparsity makes it difficult to leverage performance gains on GPUs. Specialized hardware has been designed specifically to deploy networks that have undergone unstructured pruning (Han et al., 2015). In SNNs, because event-driven hardware triggers computations only when both incoming spikes and weights are non-zero, SNNs can better leverage the unstructured sparsity brought about by their event-driven characteristics (Merolla et al., 2014; Chen et al., 2022). Unstructured pruning for SNNs remains an important area of research, with many studies conducted in this regard (Kim et al., 2022; Yin et al., 2023; Chen Y. et al., 2023; Han et al., 2022).

In the context of network pruning, the Lottery Ticket Hypothesis (LTH) (Frankle and Carbin, 2018) suggests that within densely and randomly initialized feed-forward neural networks, there exist subnetworks that can achieve performance comparable to the original network within a similar number of iterations when trained independently. These subnetworks are referred to as “winning tickets”. In the domain of ANN pruning, a series of related work has achieved notable results. Supermasks that encode strong inductive biases are far superior to random masks (Zhou et al., 2019). The significance of the initial state of winning tickets has prompted the use of a “rewinding” mechanism to ensure stability in the training process (Frankle et al., 2019). The discovery that neural networks transition from learning low-frequency to high-frequency components during optimization has led to the concept of “early-bird” lottery tickets in network pruning (Tanaka et al., 2020). In the SNN domain, the LTH has also been analyzed and applied (Kim et al., 2022). However, several challenges remain in the SNN domain, such as how to evaluate network connections in SNNs to identify optimal winning tickets, how to design pruning strategies for redundant frequency-encoded components in temporal datasets, and how to achieve higher sparsity while minimizing accuracy loss for hardware deployment. Our paper will propose a pruning algorithm from these perspectives.

In this paper, we propose a spatio-temporal pruning algorithm tailored for SNNs. Our approach optimizes network efficiency by accounting for sequential data with temporal features along with the spatial structure of the network. We address the challenge of imbalanced pruning across different layers in SNNs which can introduce bottlenecks at high-traffic layers when processing data asynchronously. To address these challenges, we propose the following: (1) Applying the Layer-Adaptive Magnitude-based Pruning Score (LAMPS) (Lee et al., 2020) technique to SNNs to adjust the pruning scale across layers. This approach helps reduce model distortion caused by weight magnitude biases. LAMPS calculates a layer-specific pruning score based on the connection density and weight magnitudes, promoting a balanced reduction in network complexity with minimal performance degradation. (2) For datasets that include temporal features, we address potential redundancy across time by adjusting the SNN's adaptive temporal dynamics. Our contributions can be summarized as follows:

1. We propose a spatio-temporal pruning algorithm for SNNs, which dynamically reduces both spatial and temporal redundancy. The method integrates adaptive temporal pruning and LAMPS-based layer-wise balanced spatial pruning to achieve high sparsity with minimal performance loss.
2. We analyze the relationship between KL-Divergence of neuron outputs and model parameter changes, demonstrating the effectiveness of adaptive temporal dynamics in optimizing pruning decisions.
3. Experimental results show that a 98% reduction in parameter space across all four datasets. An improvement in accuracy is obtained on the two DVS datasets with time-varying features, upon conducting a 50% and 20% reduction in time redundancy, respectively.

## 2 Related works

In this section, we present the progress and background of pruning algorithms in the field of SNNs. We provide a detailed introduction to the relevant concepts utilized, such as the LAMPS and how event-camera datasets are integrated into the pruning process.

### 2.1 Spiking neural network pruning

Spiking Neural Networks (SNNs) leverage dynamical sparsity, while pruning enhances static sparsity, leading to extensive exploration in combining these complementary features. Methods derived from Artificial Neural Networks (ANNs) have been adapted to SNNs, such as the work by Deng et al. (2021), which employs the Alternating Direction Method of Multipliers (ADMM) optimization with sparsity regularization to compress SNNs. Chen et al. (2022) proposed a pruning strategy that regulates pruning speed by modifying the threshold function of state transitions, enabling more effective sparsification. Chowdhury et al. (2021, 2022) introduced a method for pruning the temporal dimension of SNNs by analyzing the principal components of the accumulated membrane potential layer by layer. Recent trends in the SNN community favor extreme, unstructured sparsity over structured pruning, as unstructured sparsity is more compatible with neuromorphic hardware (Meng et al., 2023). Kim et al. (2022) explored the lottery ticket hypothesis in SNNs and implemented the Iterative Magnitude Pruning (IMP) method, demonstrating that high sparsity can be achieved while maintaining accuracy. Some methods also integrate neuron states to prune both neurons and synaptic weights (Han et al., 2024; Shi et al., 2024a; Han et al., 2025). However, despite advancements in combining dynamical and static sparsity, the full potential of spatio-temporal pruning remains largely untapped. Our approach is inspired by biological synaptic pruning, a key mechanism in human cognitive development, where redundant synapses are selectively eliminated to enhance brain efficiency (Fleming and McDermott, 2024). This process is crucial for attention (Das et al., 2024), cognitive control (Millán et al., 2019), and memory optimization (Faust et al., 2021), all of which align with the objectives of artificial neural network pruning. Furthermore, spatio-temporal optimization techniques have been extensively studied in deep reinforcement learning (DRL) (Rühling Cachay et al., 2023; Cao et al., 2020) and biological network modeling (Lin et al., 2021; Delasalles et al., 2019). By incorporating similar principles, our method seeks to enhance computational efficiency while preserving task performance.

Conventional ANN pruning techniques, including structured and unstructured weight pruning, primarily aim to reduce model size while maintaining accuracy. However, these methods do not account for the event-driven and temporally dynamic characteristics of SNNs (Eshraghian et al., 2021). Our approach extends beyond static weight sparsity by introducing a temporal pruning component that adaptively removes redundant timesteps based on KL-divergence, a technique not present in traditional ANN pruning. Additionally, while ANN pruning typically applies fixed pruning rates across layers, our method dynamically adjusts pruning based on inter-layer weight distributions using LAMPS, ensuring that critical layers retain sufficient connectivity. By integrating both static and dynamic sparsity mechanisms, our spatio-temporal pruning algorithm effectively reduces the parameter space and computational cost of SNNs without compromising accuracy, thus bridging the gap between biologically inspired principles and efficient artificial neural network design.

### 2.2 Layer-adaptive magnitude-based pruning score

Following the insights on global and hierarchical pruning scales for SNNs (Kim et al., 2022), we choose to employ global pruning. However, our approach differs from direct global pruning, in that it first evaluates the significance of connections within layers before introducing inter-layer information. We introduce the LAMPS (Lee et al., 2020) technique from ANNs into SNNs to score the synaptic weights. For a feed-forward network with depth *d*, the synaptic weights can be denoted as *W*^(1)^, ⋯ , *W*^(*i*)^, ⋯ , *W*^(*d*)^. We unfold each layer in the *d*-layer network into a one-dimensional tensor and sort them in ascending order. For each synaptic weight in these layers, LAMPS is applied as follows:


(1) $$score(u^{\left(i\right)}):=\frac{{\left(W^{\left(i\right)}[u^{\left(i\right)}]\right)}^{2}}{\sum_{v^{\left(i\right)}\geq u^{\left(i\right)}}{\left(W^{\left(i\right)}[v^{\left(i\right)}]\right)}^{2}}$$

where *u*^(*i*)^ and *v*^(*i*)^ represent the weights of two connections after flattening the *i*-th layer, with *v*^(*i*)^ > *u*^(*i*)^. *W*^(*i*)^[*v*^(*i*)^] denotes the *v*^(*i*)^-th weight in the sorted list of *W*^(*i*)^. To prune a global *p%* of connections, we can obtain the threshold for each layer and prune the parts where LAMPS score is below this threshold. Overall, this is still a global pruning strategy, but its scoring calculation incorporates intra-layer weight magnitude information. In layers with fewer connections and already strong representational capability (where a smaller number of connections leads to a smaller denominator), the LAMPS score will be higher, preventing further pruning of that layer.

### 2.3 Event camera-based datasets

The DVS128-Gesture (Li et al., 2017) dataset consists of various hand gestures in different lighting conditions and subjects captured using an event camera. The CIFAR10-DVS (Amir et al., 2017) dataset is created by capturing the CIFAR-10 dataset with an event camera, through repeated closed-loop smooth (RCLS) motion, generating rich local intensity changes. A common method for processing DVS datasets at present is to integrate events over fixed time intervals, transforming the original event stream into frame-based data (Fang et al., 2023). Firstly, we denote the Event data as *E*(*x*_*i*_, *y*_*i*_, *t*_*i*_, *p*_*i*_) and divide it evenly into *T* segments. A given frame in the integrated frame data is denoted as *F*(*j*). The value at the position (*p, x, y*) is *F*(*j, p, x, y*). The frame *F*(*j*) is obtained by integrating the events in the event stream with indices ranging between *j*_*l*_ and *j*_*r*_, where


(2) $$\begin{array}{l} j_{l}=\left\lfloor \frac{N}{T}\right\rfloor \cdot j \end{array}$$

(3) $$j_{r}=\left\{ \begin{array}{l} \left\lfloor \frac{N}{T}\right\rfloor \cdot (j+1),\text{ if }j<T-1\\ N,\text{ if }j=T-1 \end{array}\right.$$

(4) $$\begin{array}{l} F\left(j,p,x,y\right)=\overset{j_{r}-1}{\underset{i=j_{l}}{\sum }}I_{p,x,y}\left(p_{i},x_{i},y_{i}\right) \end{array}$$

where ⌊.⌋ is downward rounding. $I_{p,x,y}\left(p_{i},x_{i},y_{i}\right)$ is the indicator function, when (*p, x, y*) = (*p*_*i*_, *x*_*i*_, *y*_*i*_) takes the value 1.

## 3 Method

In this section, we demonstrate the specific implementation process and theoretical basis of the proposed spatio-temporal pruning scheme for SNNs. Firstly, we introduce the SNNs and the adopted Leaky Integrate-and-Fire (LIF) neuron model. Subsequently, we describe the framework of the proposed spatio-temporal pruning scheme. Following that, we design a unique pruning method for SNNs, with optimization functions and constraints specifically designed for it. Lastly, we present an evaluation design for the pruning algorithm, considering both the output and the variations in network parameters.

### 3.1 Spiking neural network

SNNs serve as a simulation network for the spatio-temporal dynamic behavior of biological neural circuits, distinguishing themselves from traditional ANNs primarily through neuron designs inspired by biological mechanisms (Hu et al., 2023) and their inherent capacity for temporal information representation. In this study, we focus on the LIF neuron (Hunsberger and Eliasmith, 2015), which is not only the most commonly used model in SNNs but also represents a compromise between biological complexity and computational feasibility, making it suitable for large-scale SNN simulations (Fang et al., 2023). The behavior of the classic LIF model can be modeled as follows:


(5) $$\begin{array}{l} \tau \frac{\text{d}u}{\text{d}t}=-\left(u-u_{rest}\right)+R\cdot I\left(t\right),u<V_{th} \end{array}$$

where τ and *R* are the time constant and resistance, *I*(*t*) are the input current from the pre-synaptic membrane potential. *V*_*th*_ represents the neuron membrane potential firing threshold, and *u*_*rest*_ represents the resting potential membrane potential. If the membrane potential *u* exceeds *V*_*th*_, a spike is fired and *u* is reset to *u*_*rest*_. For numerical simulations of LIF neurons, we need to consider a discrete version of the parameter dynamics. Assuming *u*_*rest*_ is 0 (Fang et al., 2023; Li et al., 2021), the spike firing function and hard reset can be expressed as:


(6) $$\begin{array}{r} a\left(t+1\right)=\text{Θ}\left(u\left(t+1\right)-V_{th}\right),\\ u\left(t+1\right)\leftarrow u\left(t+1\right)\cdot \left(1-a\left(t+1\right)\right) \end{array}$$

where Θ(·) denotes the Heaviside step function, which emits a spike when *u*(*t*+1) exceeds the threshold function. The output spike *a*(*t*+1) acts as a messenger for information propagation between network layers and affect the subsequent layer.

### 3.2 Overall architecture

**Figure 1:**
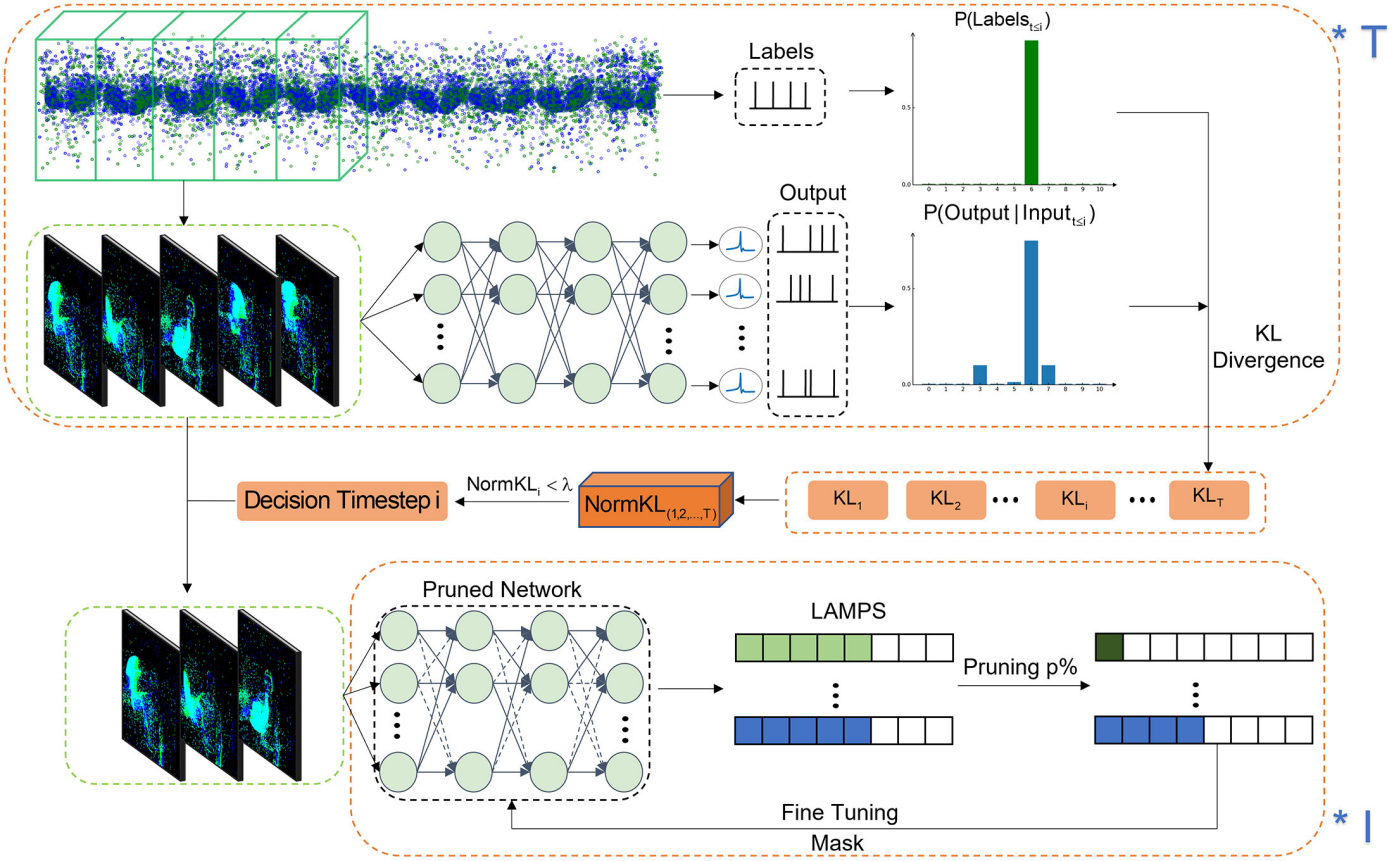
The framework of the spatio-temporal pruning algorithm. By calculating the KL-divergence of *T* different decision timesteps, the decision timestep is selected for adaptive temporal dynamics and spatial dimension pruning is performed through *I* iterations of fine-tuning.

The overall process of the proposed spatiotemporal pruning framework is illustrated in Figure 1. Given a sequential dataset such as the DVS dataset, it is initially integrated into frame-level representations using a standard event data integration operation. The frame sequence can be represented as *D* ∈ ℝ^*T*×2 × *W*×*H*^, where *T* denotes the number of frames. The frame sequence *D* is then fed into an SNN for classification, and the output layer of the network is monitored.

To determine the optimal decision timestep, we analyze the model's classification confidence across different time lengths. Specifically, we extract sequences of various lengths *D*_1~*t*_ and compute the accumulated spike responses from the output layer. To quantify the similarity between the model output and the true labels over time, we compute the KL-divergence between the predicted class probabilities and the ground-truth distribution:


(7) $$\begin{array}{l} KL\left(p\left(y_{1:t}|x_{1:t},\theta \right)||p\left(y^{*}\right)\right)=\underset{c}{\sum }p\left(y_{c,1:t}|x_{1:t},\theta \right)\log\frac{p\left(y_{c,1:t}|x_{1:t},\theta \right)}{p\left(y_{c}^{*}\right)} \end{array}$$

where *p*(*y*_1:*t*_|*x*_1:*t*_, θ) represents the model's predicted probability distribution over classes up to timestep *t*, and *p*(y^*^) denotes the one-hot encoded ground-truth label distribution. The KL divergence measures the discrepancy between these two distributions, providing an indication of the model's decision stability over time.

To facilitate threshold-based pruning, the sequence of computed KL divergences is subjected to min-max normalization:


(8) $$\begin{array}{l} {NormKL}_{t}=\frac{KL\left(p\left(y_{1:t}|x_{1:t},\theta \right)||p\left(y^{*}\right)\right)-KL_{\min}}{KL_{\max}-KL_{\min}} \end{array}$$

where *KL*_min_ and *KL*_max_ are the minimum and maximum KL divergence values observed in the sequence. We then compare this normalized KL divergence with a predefined threshold λ to determine the decision timestep *t*′:


(9) $$\begin{array}{l} t^{\prime }=\min\left\{t|{NormKL}_{t}<\text{λ}\right\} \end{array}$$

This means that we select the earliest timestep where the KL divergence stabilizes below the threshold, ensuring that the model has accumulated sufficient information for a reliable classification decision. Applying this decision timestep in the temporal dimension to the sequence data *D* yields a new sequence $D_{1\sim t^{\prime }}$.

**Figure 2:**
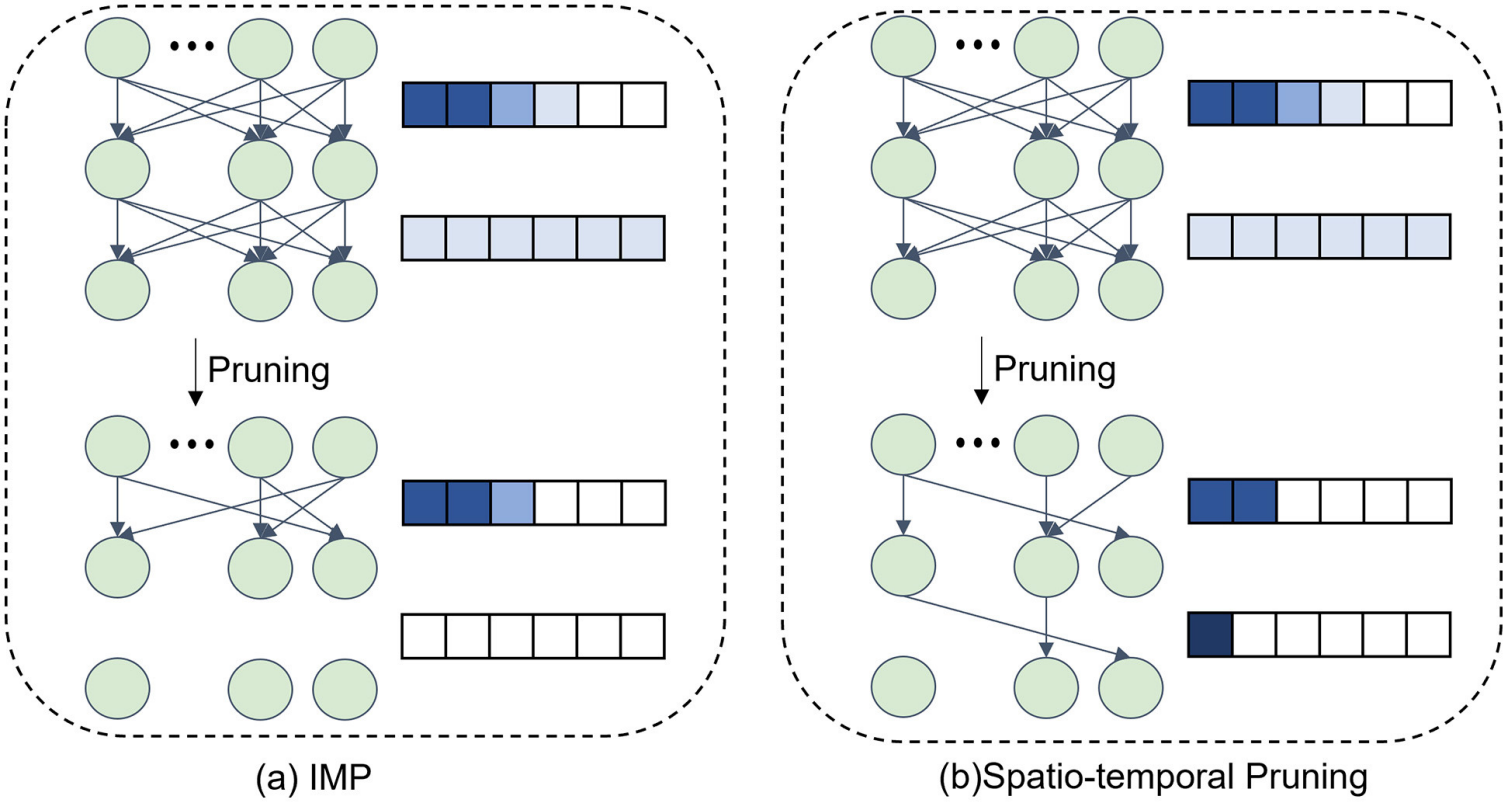
The distinction in connection selection by pruning methods. **(a)** The IMP method selects weights for pruning solely based on magnitude. **(b)** The proposed spatio-temporal pruning algorithm considers the size relationship between layers to avoid pruning layers that already have fewer parameters.

After completing the pruning in the temporal dimension, we proceed to sparsify the network weights in the spatial dimension. Since the relative importance of weights varies across layers, we leverage the LAMPS to compute the weight significance of each layer in the SNN. The synaptic weights are ranked globally, and at each pruning iteration, the least important *p%* of the weights are pruned while maintaining a balanced pruning ratio across layers. The iterative pruning process ensures that the network achieves high sparsity while preserving performance. The impact of our pruning strategy compared to the IMP (Kim et al., 2022) method is illustrated in Figure 2. Compared to IMP, which prunes connections based solely on weight magnitudes in an iterative manner, our proposed spatio-temporal pruning algorithm additionally considers inter-layer weight distribution and temporal redundancy, leading to a more balanced and efficient pruning strategy.

The full pruning procedure is detailed in Algorithm 1. The TrainSNN function utilizes the widely adopted Spatio-Temporal Backpropagation (STBP) algorithm for training, incorporating the Atan function as a surrogate gradient to handle the non-differentiability of spikes (Wu et al., 2018). The network architectures and hyperparameters used follow the configurations provided in SpikingJelly for each corresponding dataset (Fang et al., 2023).

**Algorithm 1:** Two-stage pruning framework.

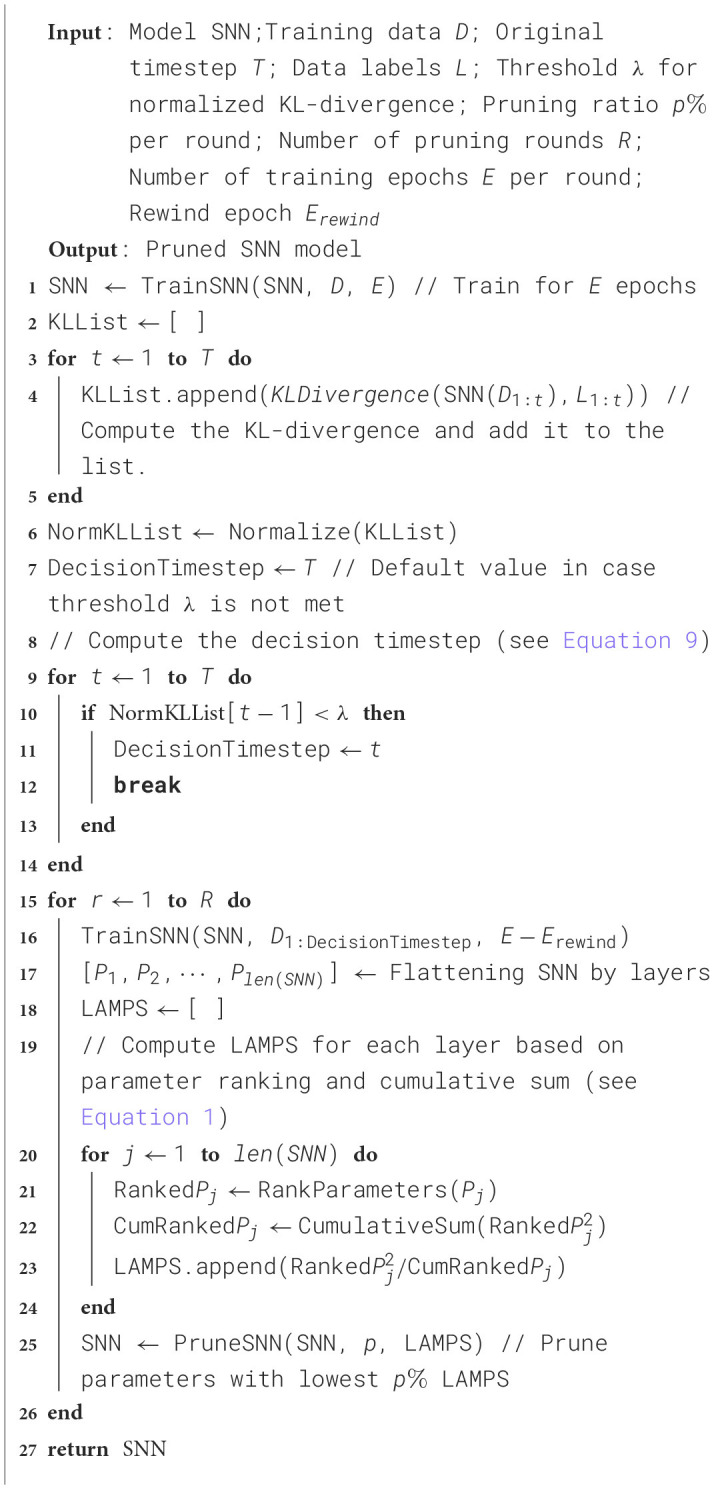

### 3.3 Optimization for sparsity

In general, the pruning methods for SNNs are constrained by the conditions of actual deployment, aiming to increase the sparsity of the network while minimizing accuracy degradation. In this section, we analyze the problem from two perspectives: the sparsity of weights in the spatial dimension and the necessity of timesteps in the temporal dimension. The pruning constraint optimization problem for SNNs is formulated as follows:


(10) $$\begin{array}{r} \arg\underset{\theta ,t}{\text{min}}\;L\left(\theta ,t\right)\\ s.t.\;\ell_{0}\left(\theta \right)\leq \ell_{0}\left(\theta^{+}\right)\\ \;t\leq T_{max} \end{array}$$

where L(·) represents the loss function of the model. θ and *t* respectively denote the optimization parameters of SNNs and the timesteps required by the model. $\ell_{0}\left(\theta^{+}\right)$ represents the L0-norm of the model parameters that meet the deployment constraints, implying that the actual deployment model parameters θ should be more sparse than θ^+^. The constraint $\ell_{0}\left(\theta \right)\leq \ell_{0}\left(\theta^{+}\right)$ ensures that the number of nonzero parameters remains within the permissible range for hardware deployment, considering factors such as limited memory bandwidth, energy efficiency, and real-time inference constraints. *T*_*max*_ represents the maximum allowable timesteps, ensuring that the computation cost remains feasible.

### 3.4 Adaptive temporal dynamics

In Algorithm 1, selecting the decision timestep (line 8-14) is a crucial step in temporal pruning. This section elaborates on how to determine the optimal classification timestep by leveraging KL-divergence. Considering the output of SNNs in specific tasks, the optimal classification should occur as early as possible while ensuring correctness. The optimization objective function is formulated as:


(11) $$\begin{array}{l} \arg\underset{\theta ,t}{\min}KL\left[p\left(y_{1:t}|x_{1:t},\theta \right);p\left(y_{1:t}|x_{1:t},\theta^{*}\right)\right] \end{array}$$

where θ^*^ represents the globally optimized network parameters that yield the best classification performance. *x*_1:*t*_ represents the input data from timestep 1 to *t*, and *y*_1:*t*_ represents the corresponding model output. It is worth noting that this can be regarded as two random variables. The probability function *p*(·) encodes the model's confidence in different classes.

To determine the optimal decision timestep, we normalize the KL-divergence sequence across different timesteps using min-max normalization:


(12) $$\begin{array}{l} {NormKL}_{t}=\frac{KL\left[p\left(y_{1:t}|x_{1:t},\theta \right);p\left(y_{1:t}|x_{1:t},\theta^{*}\right)\right]-KL_{\min}}{KL_{\max}-KL_{\min}} \end{array}$$

where *KL*_max_ and *KL*_min_ denote the maximum and minimum KL-divergence values observed across all timesteps.

We define a threshold λ for selecting the decision timestep:


(13) $$\begin{array}{l} t^{\prime }=\min\left\{t|{NormKL}_{t}<\text{λ}\right\} \end{array}$$

This ensures that we select the earliest timestep where the normalized KL-divergence falls below the threshold, indicating that the model has accumulated sufficient information for a reliable classification decision.

Given these constraints, the final optimization problem is formulated as:


(14) $$\begin{array}{l} \arg\underset{\theta ,t}{\text{min}}KL\left[p\left(y_{1:t}|x_{1:t},\theta \right);p\left(y_{1:t}|x_{1:t},\theta^{*}\right)\right]\\ \;s.t.\;\ell_{0}\left(\theta \right)\leq \ell_{0}\left(\theta^{+}\right)\\ \;t\leq T_{max}\\ \;{NormKL}_{t}<\text{λ} \end{array}$$

### 3.5 Dynamics from the information perspective

The Fisher Information Matrix (FIM) is widely used to quantify the information content of model parameters. In our case, it helps assess how much information changes across timesteps. To approximate the KL-divergence function around the optimal parameters θ^*^, we apply a second-order Taylor expansion:


(15) $$\begin{array}{l} KL\left[p\left(y_{1:t}|x_{1:t},\theta \right);p\left(y_{1:t}|x_{1:t},\theta^{*}\right)\right]\\ \approx KL{\left[p\left(y_{1:t}|x_{1:t},\theta \right);p\left(y_{1:t}|x_{1:t},\theta^{*}\right)\right]}_{\theta^{*}=\theta }\\ +\nabla_{\theta^{*}}KL{\left[p\left(y_{1:t}|x_{1:t},\theta \right);p\left(y_{1:t}|x_{1:t},\theta^{*}\right)\right]}_{\theta^{*}=\theta }^{T}\left(\theta^{*}-\theta \right)\\ +\frac{1}{2}{\left(\theta^{*}-\theta \right)}^{T}\nabla_{\theta^{*}}^{2}KL{\left[p\left(y_{1:t}|x_{1:t},\theta \right);p\left(y_{1:t}|x_{1:t},\theta^{*}\right)\right]}_{\theta^{*}=\theta }\left(\theta^{*}-\theta \right)\\ =\frac{1}{2}{\left(\theta^{*}-\theta \right)}^{T}F_{t}\left(\theta^{*}-\theta \right) \end{array}$$

where the first and second terms vanish, leaving only the Fisher Information Matrix *F*_*t*_ in the final term. This demonstrates that the KL-divergence is directly related to the FIM, reinforcing the intuition that pruning based on KL-divergence preserves informative parameters.

The FIM is essential for understanding the sensitivity of network parameters. However, directly computing the true FIM is computationally intractable due to its high dimensionality and dependence on the full dataset. In practice, the empirical FIM is used to approximate the true FIM (Singh and Alistarh, 2020; Kim et al., 2023; Kunstner et al., 2019):


(16) $$\begin{array}{l} {\hat{F}}_{t}=\frac{1}{N}\overset{N}{\underset{n=1}{\sum }}||\nabla_{\theta }\log p\left(y_{1:t}|x_{1:t},\theta \right){||}^{2} \end{array}$$

where *N* is the batch size, and ∇_θ_ represents the gradient computed during training. Since the optimization process relies on minimizing the divergence between predicted and true outputs, the empirical FIM provides a meaningful measure of parameter importance.

This establishes a direct relationship between KL-divergence-based Adaptive Temporal Dynamics and changes in model parameters, allowing for an efficient pruning strategy that dynamically adapts to both spatial and temporal constraints. This theoretical foundation justifies the decision timestep selection process in Algorithm 1 (line 8–14), ensuring that pruning decisions are made based on a principled optimization framework.

## 4 Experiments

The proposed method is evaluated on static image classification benchmarks (CIFAR-10 and CIFAR-100) as well as DVS data benchmarks (DVS128 Gesture and CIFAR10-DVS). The implementation of the pruning is based on the open-source SNN framework SpikingJelly (Fang et al., 2023). Firstly, we explain the detailed settings and experimental environment. Then, we conduct ablation experiments on the temporal and spatial modules of the proposed spatio-temporal pruning algorithm, calculating the performance of the ablated modules and some evaluation metrics. Finally, we perform experimental statistics on the neuron firing rates and the model's synaptic weight connections before and after the use of the spatio-temporal pruning algorithm.

### 4.1 Settings

We use four datasets in our experiments: CIFAR10/100, DVS128-Gesture, and CIFAR10-DVS. The basic information of each dataset and the network architectures used are shown in Table 1. The experiments are conducted on an Ubuntu 20.04 LTS system with two NVIDIA RTX 4090 GPUs. The specific parameters and training pipeline settings are as follows:

**Table 1:** The basic information of the datasets and the corresponding structures.

**Dataset**	**Image**	**Training samples**	**Testing samples**	**Category**	**Structure**	**Surrogate function**
CIFAR10	32 × 32 × 3	50,000	1,000	10	ResNet19	ATan
CIFAR100	32 × 32 × 3	50,000	10,000	100	VGG16 & ResNet19	ATan
DVS128-Gesture	128 × 128 × 2	1,176	288	11	5Conv, 2FC	ATan
CIFAR10-DVS	128 × 128 × 2	10,000	1,000	10	4Conv, 2FC & VGGSNN	ATan

#### 4.1.1 CIFAR-10/100

For the CIFAR-10 dataset, we use the ResNet19 network. For the CIFAR-100 dataset, we use both the VGG16 and ResNet19 networks, following previous work (Kim et al., 2022). The first convolutional layer converts static images into spike form. Data augmentation techniques such as RandomCrop, RandomHorizontalFlip, Cutout, and Normalize are applied (Kim et al., 2022). We set the batch size to 128 and the timestep to 5. The models are trained using the SGD optimizer with a learning rate of 0.3, momentum of 0.9, and weight decay of 0.0005. The cosine learning rate scheduling strategy is adopted.

#### 4.1.2 DVS128-Gesture

For the DVS128Gesture dataset, we use the official implementation from SpikingJelly (Fang et al., 2023). The batch size is set to 8 and the timestep to 20. The Adam optimizer is used with a learning rate of 0.001. The surrogate gradient function is the Atan function with a parameter α of 2.0. The hyperparameter λ is set to 0.01, and the cosine learning rate scheduling strategy is used.

#### 4.1.3 CIFAR10-DVS

We adopt the official implementation from SpikingJelly (Fang et al., 2023) with a 4Conv, 2FC network. Under this network architecture, the batch size is set to 16. Additionally, we implement the VGGSNN network (Shi et al., 2024a), where we follow the same TET loss (Deng et al., 2022) and data augmentation (Li et al., 2022; Shi et al., 2024a) strategies as in Shi et al. (2024a), with the batch size set to 64. In both network architectures, the timestep is set to 10. The model is trained using the Adam optimizer with a learning rate of 0.001. Similar to the DVS128Gesture dataset, the surrogate gradient function is Atan with α = 2.0, and the hyperparameter λ is set to 0.01. The cosine learning rate scheduling strategy is applied.

### 4.2 Performance

Table 2 demonstrates the performance variations of our spatial pruning algorithm on static images at different levels of sparsity. It is evident that our method can maintain good performance while achieving higher sparsity. On the CIFAR10 dataset, when sparsity reaches 98.13%, it can still maintain a high accuracy rate (with a reduction of about 0.18%). On the larger CIFAR100 dataset, we compared two network architectures. Our method outperforms the comparison methods at higher levels of sparsity. On ResNet19, when the sparsity rate reaches 98.13%, our method exhibits very little loss in accuracy, which is a significant improvement (greater than 1%) compared to the comparison algorithm (Kim et al., 2022).

**Table 2:** Comparison with other algorithms on static datasets.

**Dataset**	**Pruning method**	**Structure**	**Base acc(%)**	**Sparsity(%)**	**Param. (M)**	**ΔAccuracy(%)**
CIFAR10	SCCD-SNN (Meng et al., 2023)	ResNet20	92.14	70.00	1.30	−2.78
	Deep R (Bellec et al., 2017)	ResNet19	93.22	94.25	0.73	−1.31
				97.56	0.31	−2.10
	Grad R (Chen et al., 2021)	6 Conv, 2 FC	92.84	97.65	0.86	−1.47
				99.27	0.26	−3.52
	IMP (Kim et al., 2022)	ResNet19	93.22	97.54	0.31	−0.04
				98.13	**0.23**	−0.79
	Ours	ResNet19	92.40	96.75	0.41	**0.42**
				97.54	0.31	**0.34**
				98.13	**0.23**	**−0.18**
CIFAR100	Grad R (Chen et al., 2021)	ResNet19	71.34	94.92	0.64	−3.87
				97.65	0.30	−4.03
	IMP (Kim et al., 2022)	VGG16	69.19	96.75	0.48	−1.84
				97.54	0.36	−2.31
				98.13	0.27	−3.34
		ResNet19	71.34	95.69	0.55	−0.89
				97.54	0.31	−2.29
				98.13	**0.24**	−3.99
	Ours	VGG16	69.19	96.75	0.48	**−1.12**
				97.54	0.36	**−1.65**
				98.13	0.27	**−2.86**
		ResNet19	71.58	95.69	0.55	**−0.68**
				97.54	0.31	**−2.01**
				98.13	**0.24**	**−2.86**

Bold values indicate cases where the parameter count is relatively low or the accuracy degradation is minimal, demonstrating efficient pruning results.

The performance of our model under different levels of sparsity on the DVS dataset and the numerical values of accuracy degradation are shown in Table 3. The “+S” indicates the results after applying spatial pruning strategies, and “+T” represents the outcomes after incorporating the Adaptive Temporal Dynamics strategy. “*/*” represents the decision timestep and total timestep of Adaptive Temporal Dynamics. For example, “10/20” indicates that the model originally needs to calculate 20 timesteps, but the decision timestep is set to 10. After performing spatial pruning on the DVS128-Gesture dataset, our model was able to achieve good performance at higher sparsity levels. The performance started to decline when the Pruning rate exceeded 98%. Surprisingly, after adding Adaptive Temporal Dynamics, by training and inferring in the temporal dimension with a reduced decision timestep from 20 to 10 (which directly halves the synaptic operations), the model's performance improved. On the CIFAR10-DVS dataset, our model still maintained good performance at high sparsity levels (greater than 98%) after spatial pruning. With the introduction of Adaptive Temporal Dynamics, the decision timestep used for training and inference in the temporal dimension was reduced from 10 to 8, and the model still achieved excellent performance.

**Table 3:** Comparison with other algorithms on the DVS datasets.

**Dataset**	**Pruning method**	**Structure**	**Base acc (%)**	**Sparsity (%)**	**Param. (M)**	**Accuracy (%)**	**ΔAccuracy (%)**
DVS128-Gesture	STDS (Chen et al., 2022)	5Conv, 2FC	95.83	94.50	0.094	94.44	−1.39
				96.90	0.053	92.36	−3.47
				97.30	0.046	83.68	−12.15
	IMP (Kim et al., 2022)	5Conv, 2FC	95.83	96.72	0.056	91.32	−4.51
				97.51	0.042	86.11	−9.72
				98.11	0.032	78.47	−17.36
	SCCD-SNN (Meng et al., 2023)	8Conv, 1FC	94.44	20.00	0.136	94.44	0.00
				50.00	0.085	91.67	−2.77
				90.00	**0.017**	75.35	−19.09
	Our(+S)	5Conv, 2FC	95.83	96.72	0.056	**95.83**	**0.00**
				97.51	0.042	**96.18**	**0.35**
				98.11	0.032	95.13	−0.70
	Our(+S+T 10/20)	5Conv, 2FC	95.83	96.72	0.056	**95.83**	**0.00**
				97.51	0.042	**96.18**	**0.35**
				98.11	0.032	**96.52**	**0.69**
CIFAR10-DVS	STDS (Chen et al., 2022)	4Conv, 2FC	71.70	84.12	0.745	71.37	−0.33
				95.29	0.221	69.05	−2.65
				96.20	0.178	64.92	−6.78
	IMP (Kim et al., 2022)	4Conv, 2FC	71.70	96.80	0.150	70.70	−1.00
				97.59	0.113	68.00	−3.70
				98.18	**0.085**	63.30	−8.40
	SCCD-SNN (Meng et al., 2023)	8Conv, 1FC	72.60	20.00	0.952	73.60	1.00
				50.00	0.595	71.04	−1.56
				90.00	0.119	66.64	−5.96
	TEE-SNN (Shi et al., 2024a)	VGGSNN	82.50	93.20	0.658	81.90	-0.5
				95.54	0.432	81.00	−1.40
				98.73	**0.123**	79.00	−3.40
	Our(+S)	4Conv, 2FC	71.70	96.8	0.150	72.48	0.78
				97.59	0.113	**72.78**	**1.08**
				98.18	**0.085**	**72.28**	**0.58**
	Our(+S+T 8/10)	4Conv, 2FC	71.70	96.80	0.150	**72.88**	**1.18**
				97.59	0.113	72.58	0.88
				98.18	**0.085**	71.98	0.28
	Our(+S+T 8/10)	VGGSNN	82.50	94.31	**0.551**	**83.7**	**1.20**
				95.72	**0.414**	**83.20**	**0.70**
				98.61	0.135	81.50	−1.00

Bold values indicate cases where the parameter count is relatively low or the accuracy degradation is minimal, demonstrating efficient pruning results.

In comparison with other methods, our spatial strategy exhibited lower performance degradation under high sparsity conditions in both static datasets, as shown in Table 2. Our spatiotemporal strategy was successful in the DVS dataset, achieving outstanding results in the CIFAR10-DVS dataset derived from static data (Li et al., 2017). In the DVS128-Gesture dataset, which contains richer temporal information captured by DVS cameras (Shi et al., 2024b; Anumasa et al., 2024), our strategy achieved remarkable performance improvements compared to other methods, as illustrated in Table 3.

### 4.3 Ablation study

We conduct ablation experiments on spatio-temporal pruning algorithms on two DVS datasets as shown in Table 4. The network architectures used were still 5Conv, 2FC and 4Conv, 2FC, respectively. The variables were the presence or absence of spatial and temporal strategies. We compared the model on five metrics: number of parameters, sparsity, synaptic operations (SOPs), accuracy, and decision timestep. SOPs is the number of spike-based AC operations (Chen G. et al., 2023; Zhou et al., 2023). $SOPs=\underset{i}{\sum }s_{i}c_{i}$ denotes the total number of synaptic operations. For each presynaptic neuron *i* in an SNN, *s*_*i*_ denotes the number of spikes fired by this neuron, and *c*_*i*_ denotes the number of synaptic connections from this presynaptic neuron.

**Table 4:** Ablation experiments on different datasets using temporal and spatial pruning algorithms.

**Dataset**	**Spatial**	**Temporal**	**Accuracy (%)**	**Sparsity (%)**	**Decision timestep**	**Param. (M)**	**SOPs (M)**
**DVS128-Gesture**			**95.83**	**0**	**20**	**1.70**	**601.52**
	✓		95.83	98.11	20	0.03	30.62
		✓	96.18	0	10	1.70	348.82
	✓	✓	96.52	98.11	10	0.03	15.41
CIFAR10-DVS			71.70	0	10	4.69	579.08
	✓		72.28	98.18	10	0.08	62.57
		✓	71.88	0	8	4.69	479.54
	✓	✓	71.98	98.18	8	0.08	53.93

The ablation study in Table 4 revealed that our spatio-temporal pruning scheme could reduce synaptic operations when using either the temporal or spatial strategies alone. For the spatial strategy, higher sparsity levels often directly dictate the change in the number of parameters, thereby reducing model computations. For the temporal strategy, the computed decision timestep can decrease the number of steps required for the model to make a decision, thus reducing the model's synaptic operations. The changes in accuracy indicate that both temporal and spatial strategies achieved good performance and low synaptic operations separately. Combining the temporal and spatial strategies often yields a model with even lower synaptic operations while maintaining good performance.

### 4.4 Firing rate and connectivity

**Figure 3:**
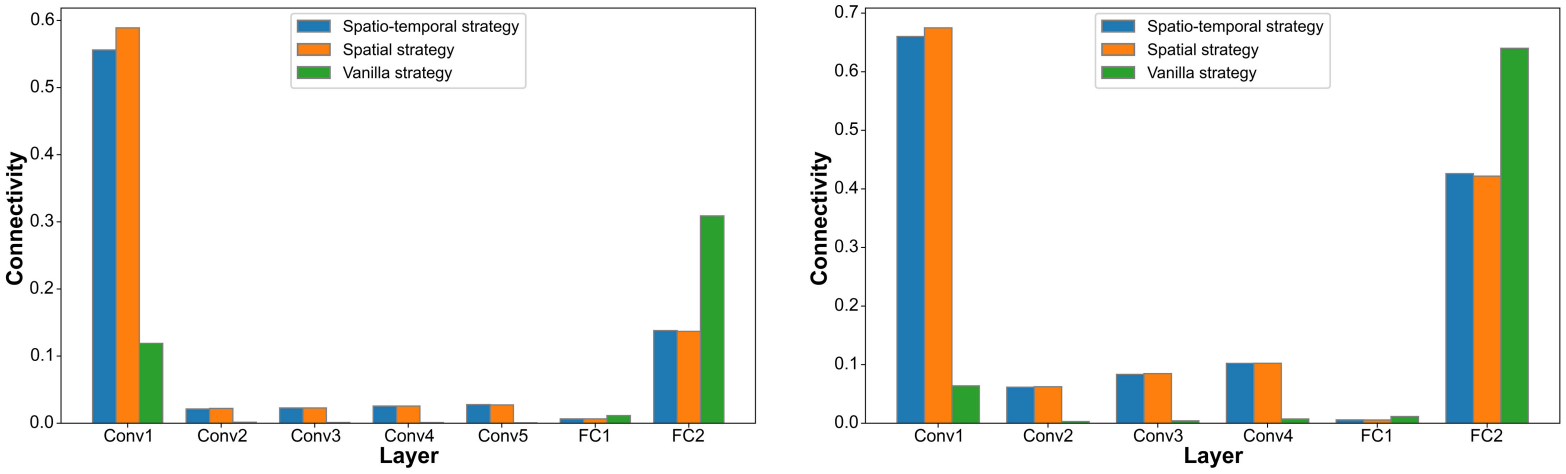
Statistics on the connection rate distribution across different layers under the Vanilla IMP strategy, spatial pruning strategy, and spatiotemporal pruning strategy on different datasets (DVS128 Gesture on the **left**, CIFAR10-DVS on the **right**).

**Figure 4:**
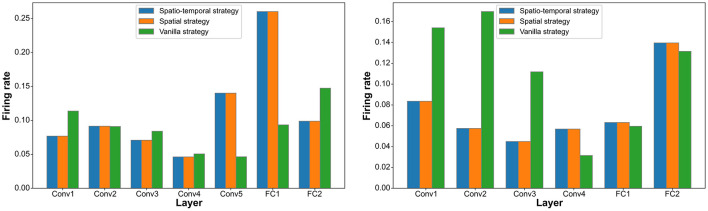
Statistics on the neuron firing rate distribution across different layers under the Vanilla IMP strategy, spatial pruning strategy, and spatiotemporal pruning strategy on different datasets (DVS128 Gesture on the **left**, CIFAR10-DVS on the **right**).

In the DVS128 Gesture and CIFAR10-DVS datasets, we analyze the connectivity and firing rates of different layers under the spatiotemporal pruning strategy, spatial pruning strategy, and Vanilla IMP strategy. As shown in Figures 3, 4, our proposed spatiotemporal pruning method tends to maintain a higher connectivity rate in shallower layers while reducing the number of connections in deeper layers. This facilitates feature extraction, as the connectivity in shallow layers often plays a more decisive role in network performance, and the feature extraction capability of shallow layers typically determines that of deeper layers. Additionally, since fully connected layers account for a significant proportion of the network parameters, our method tends to apply a more aggressive pruning strategy to them. Specifically, as shown in Figure 3, the spatiotemporal pruning strategy applies more targeted pruning to the FC1 layer, which has a higher proportion of total connections, while retaining more connections in the Conv1 layer, which has fewer total connections but plays a more crucial role. This benefit stems from the pruning strategy's ability to better estimate the remaining connection ratio for each layer. As shown in Figure 4, it can also be observed that fewer connections generally correspond to a higher neuronal firing rate, compensating for the representational capacity loss caused by weight pruning.

## 5 Analysis and discussion

In this section, we analyze the proposed spatio-temporal pruning algorithm. Firstly, we conduct a parameter analysis on the hyperparameters proposed by the model. Secondly, we analyze the experimental results brought about by Adaptive Temporal Dynamics. Then, we examine the impact of the model's total timesteps on the pruning algorithm. Finally, we conduct a statistical analysis of the magnitude of the model parameters and analyze the limitations of the algorithm.

### 5.1 Parameter analysis

**Figure 5:**
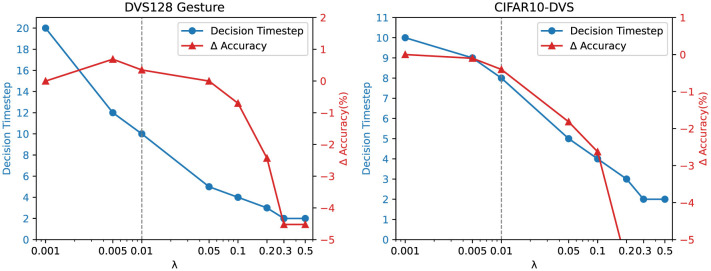
Impact of different λ values on decision timestep and accuracy across datasets.

We conducted an experimental analysis on the hyperparameter λ used in this paper, as shown in Figure 5. The hyperparameter λ serves as a threshold for the normalized KL-divergence, controlling the decision timestep in Adaptive Temporal Dynamics. A higher λ leads to a shorter inference time, as the decision timestep is reduced, allowing the model to make earlier classification decisions. Conversely, a lower λ results in a longer inference time, as the model waits for more timesteps to accumulate information before making a final decision, which may improve accuracy.

To explore this trade-off, we evaluated different values of λ on the DVS128 Gesture and CIFAR10-DVS datasets, analyzing their impact on both decision timestep and accuracy. As illustrated in Figure 5, increasing λ generally decreases the decision timestep, reducing computational cost. However, beyond a certain threshold (e.g., λ = 0.01), the accuracy gain becomes marginal, indicating diminishing returns from allowing additional timesteps.

Based on these observations, we select λ = 0.01 as the default threshold, as it provides a balance between maintaining high accuracy and reducing inference time. This choice ensures that the model reaches a stable classification decision while avoiding unnecessary computations. By tuning λ, different trade-offs between accuracy and efficiency can be achieved depending on application requirements.

### 5.2 Analysis of the results of adaptive temporal dynamics

From Table 3, it is evident that by applying Adaptive Temporal Dynamics, the obtained decision timestep on the DVS128 Gesture and CIFAR10-DVS datasets is 10 and 8, respectively. This result enables us to achieve decision timesteps that are 50% and 80% of the original ones, effectively saving about 50% and 20% of computation on the temporal dimension. After spatial pruning, the resulting sparse network performance on both datasets is comparable to that of the unpruned network. This indicates that our spatiotemporal pruning scheme can achieve higher sparsity and maintain accuracy while reducing synaptic operations.

**Figure 6:**
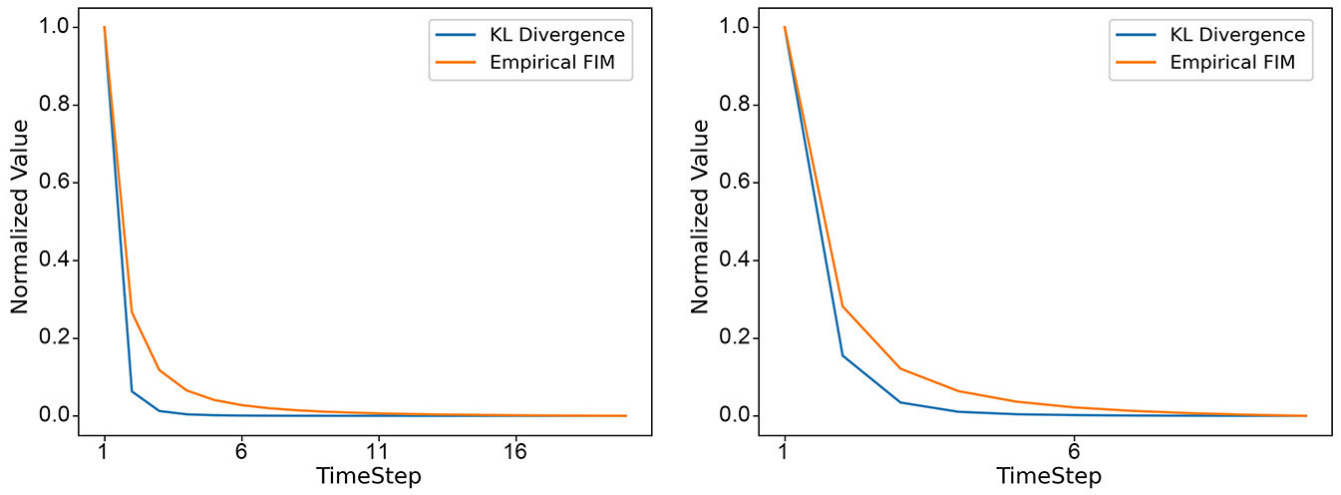
On different datasets (DVS128 Gesture on the **left**, CIFAR10-DVS on the **right**), the changes in Norm-KL and empirical FIM with the selection of different decision timesteps.

After training the model until convergence without any pruning, we employed the KL-divergence between the output layer's response at different timesteps after feeding in the training set and the true labels as an intuitive metric. Subsequently, we computed the normalized KL-divergence for different timesteps through min-max normalization. To demonstrate the efficacy of this normalized KL-divergence in representing the information content across the temporal dimension, we calculated the information encapsulated by the model at different decision timesteps, which corresponds to the values of the empirical Fisher Information Matrix (FIM). We compared the trends of these two metrics, as shown in Figure 6, and found that their variations are almost identical. This indicates that the trend of changes in the model's outputs aligns with the trend of changes in the model parameters during the learning process. This further suggests that Adaptive Temporal Dynamics is meaningful for the selection of decision timestep.

### 5.3 Analysis of the total timestep

We also analyzed the time step used for the employed SNNs, setting the total timesteps to 10, 15, 20, and 25 respectively for parameter experiments on the DVS128 Gesture dataset. The performance of the baseline model, as well as the models employing Adaptive Temporal Dynamics and spatiotemporal pruning at different timesteps, is compared and presented in Table 5. As can be observed, our pruning method maintains stability under various time step conditions and often achieves an earlier decision timestep, thereby saving a relatively larger amount of time when more steps are used. In terms of temporal consumption, our method directly saved 20%, 40%, 50%, and 60% of the time when the timesteps were set to 10, 15, 20, and 25, respectively. This further illustrates that for the classification of DVS datasets, it is not necessary to complete the computation of all time dimensions. Correct classification results can be obtained through a portion of the input, which is also due to the information partition problem caused by the integration mechanism of DVS.

**Table 5:** Experimental results on the impact of different timesteps on the pruning algorithm.

**TimeStep**	**Temporal**	**Spatial**	**Decision timestep**	**Sparsity(%)**	**Accuracy(%)**
10			10	0	94.09
	✓		8	0	94.44
	✓	✓	8	98.11	92.01
15			15	0	95.48
	✓		9	0	95.83
	✓	✓	9	98.11	95.48
20			20	0	95.83
	✓		10	0	96.18
	✓	✓	10	98.11	96.52
25			25	0	95.48
	✓		10	0	95.83
	✓	✓	10	98.11	95.48

### 5.4 Statistical analysis of model parameter magnitudes

**Figure 7:**
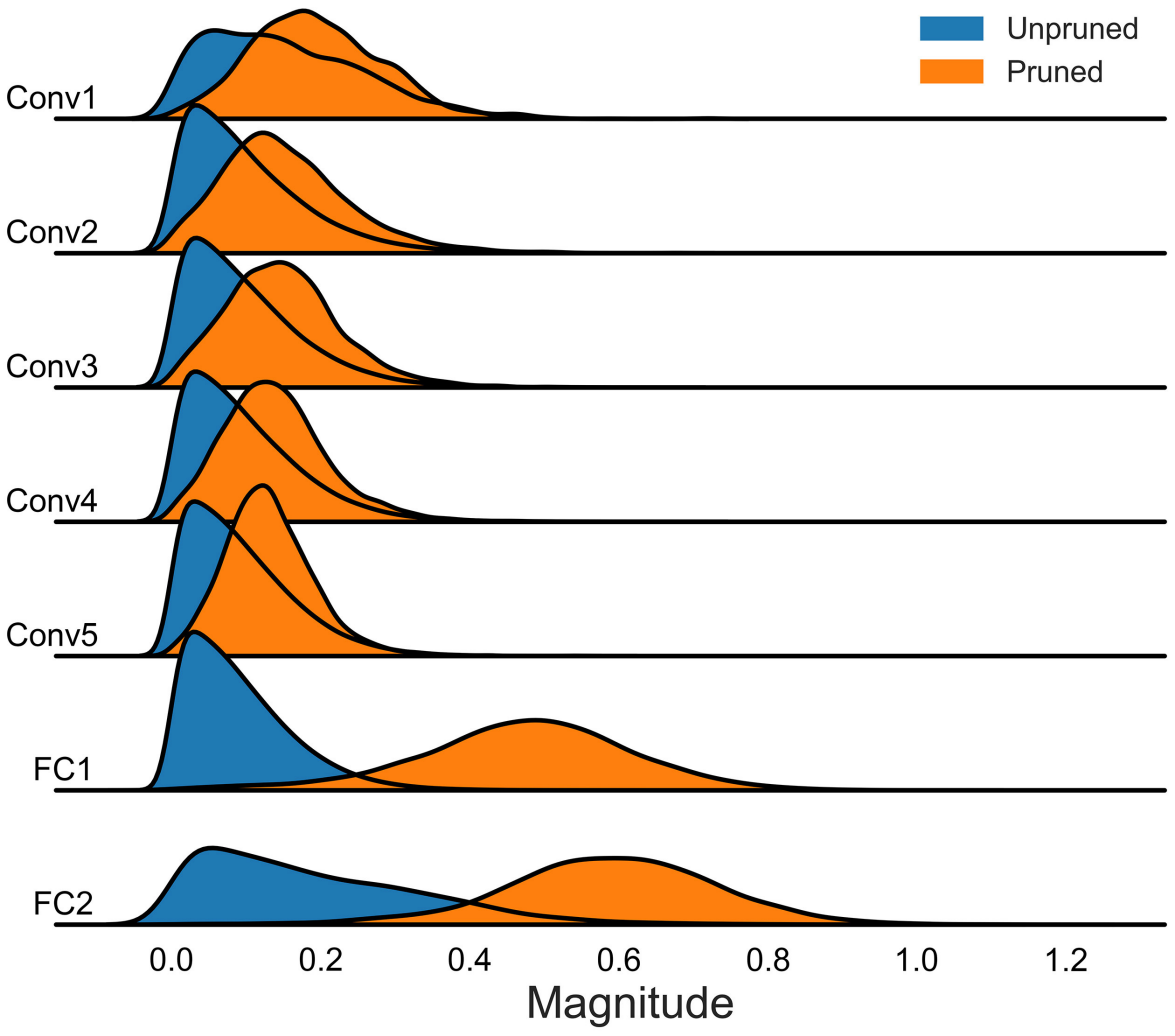
The distribution of model parameter magnitudes without pruning and after spatiotemporal pruning on DVS128 gesture.

**Figure 8:**
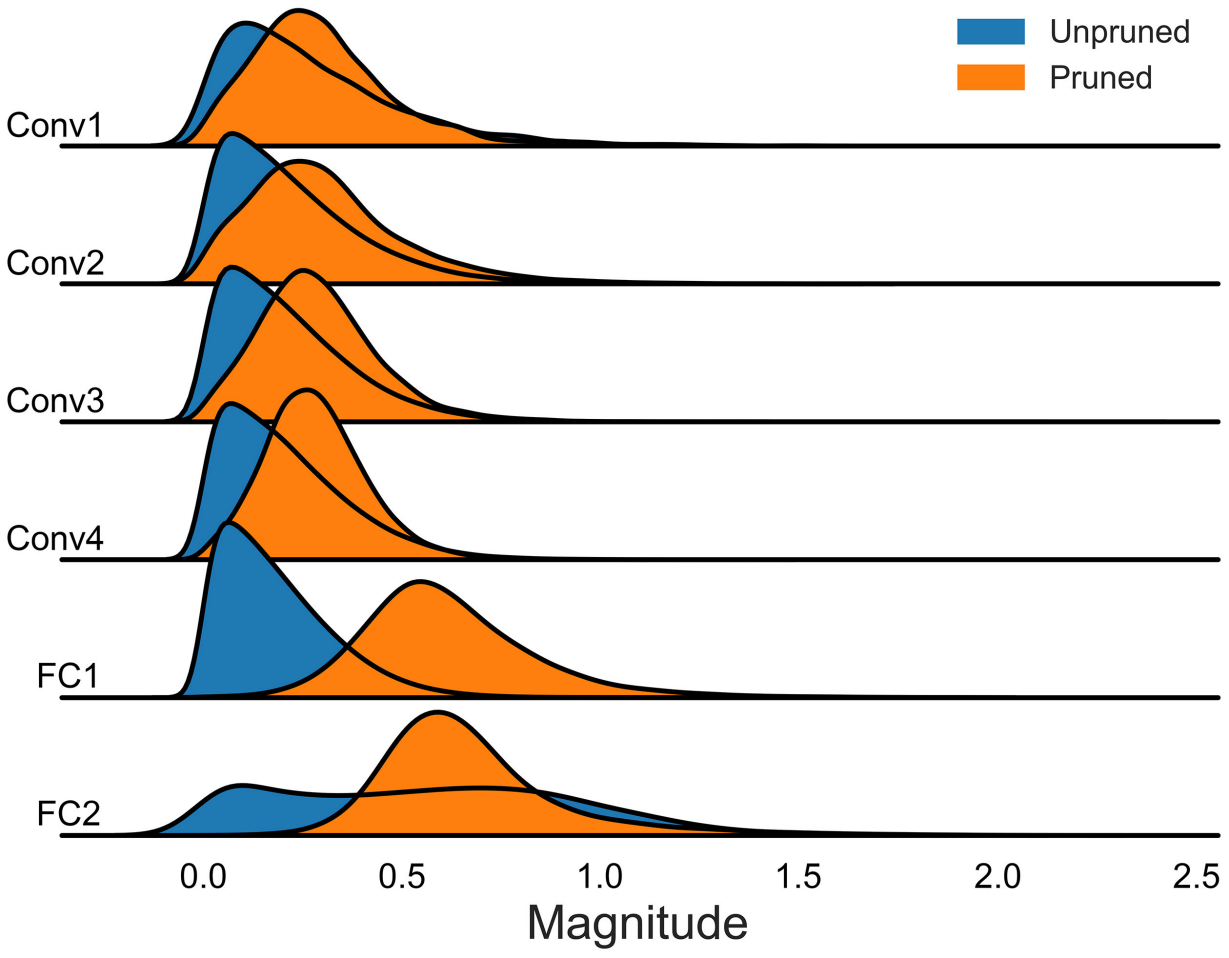
The distribution of model parameter magnitudes without pruning and after spatiotemporal pruning on CIFAR10-DVS.

We further conducted a statistical analysis of the amplitude of model parameters. This analysis was performed on models using the DVS128 Gesture and CIFAR10-DVS datasets, focusing on both the unpruned baseline models and the sparse models after spatiotemporal pruning. The results, as illustrated in Figures 7, 8, indicate that our spatiotemporal pruning algorithm tends to remove weights that have a minimal impact on the output. Consequently, the overall amplitude of the weights in the sparse model is relatively larger compared to those in the unpruned baseline model.

### 5.5 Discussion of limitations

When developing pruning algorithms for SNNs, it is crucial to consider strategies that address temporal information more thoroughly than for ANNs. Our spatio-temporal pruning algorithm is particularly effective for datasets with rich temporal information, especially those captured by raw DVS cameras. The temporal information in SNNs depends on the accumulation of membrane potential and spike generation, which is less pronounced in static datasets compared to temporal datasets. Therefore, our strategy is more suited for temporal data, emphasizing an important characteristic where SNNs focus more on neuron dynamics compared to ANNs. Future work can explore hardware-aware pruning techniques to further minimize overhead. Additionally, while our method is designed for classification tasks, extending it to detection or generative tasks is worth exploring. These tasks involve more complex temporal dependencies, which may require adapting our pruning strategy.

Beyond algorithmic considerations, our pruning approach could offer potential benefits for neuromorphic processors such as Speck (Richter et al., 2023) and Loihi (Davies et al., 2018). The Speck Development Kit, which utilizes an Address Event Representation protocol, naturally benefits from the unstructured sparsity generated by our method, reducing memory access without requiring additional hardware modifications. Additionally, the adaptive temporal pruning lowers the number of SOPs, making it highly efficient for low-power, always-on edge AI applications (Yao et al., 2024). Similarly, Loihi's event-driven architecture can leverage the reduction in redundant spikes and synaptic activations, further optimizing computational efficiency.

## 6 Conclusion

Given the inherent spatio-temporal information processing capabilities of SNNs and the resource constraints of neuromorphic hardware, efficient pruning across both the spatial and temporal dimensions of SNNs is a critical issue. In this paper, we present a spatio-temporal pruning algorithm framework tailored for the information dimensions of SNNs. For the spatial dimension, we perform a global assessment of connections by combining inter-layer weight information of SNNs, ensuring that pruning of a particular layer is not excessive. In the temporal dimension, we choose the decision timestep by comparing the model's output with the labels. We propose a pruning scheme for the temporal dimension of SNNs and link it with the Fisher Information Matrix, allowing us to evaluate the temporal pruning scheme from both the perspectives of the network's output and the network parameters' information. Our method has achieved commendable performance on several datasets tested. Especially, we have demonstrated the potential for compression in the temporal characteristics of SNNs.

However, scalability to more complex datasets and larger SNN architectures remains an open challenge. A key direction for future work is to refine the quantification of both weight importance and timestep significance to ensure balanced sparsity across different network structures. Additionally, better integration of spatial and temporal pruning is necessary to prevent undesired trade-offs, such as excessive weight pruning leading to unstable temporal dynamics. Moreover, while our approach adopts a frame-based strategy, fully asynchronous event-triggered processing remains a challenge due to current hardware constraints and is an important direction for future research.

## Data Availability

The employed datasets are publicly available online. Code is available at github.com/gzxdu/SNN_Pruning.
